# Interaction of NF-κB and FOSL1 drives glioma stemness

**DOI:** 10.1007/s00018-024-05293-1

**Published:** 2024-06-10

**Authors:** Vanajothi Ramar, Shanchun Guo, Breanna Hudson, Azam Khedri, Alyssa A. Guo, Jason Li, Mingli Liu

**Affiliations:** 1 Department of Microbiology, Biochemistry & Immunology, Morehouse School of Medicine, Atlanta, USA; 2 Department of Chemistry, Xavier University, 1 Drexel Dr, New Orleans, LA USA; 3 Wake Forest University School of Medicine, 475 Vine Street, Winston-Salem, NC USA

**Keywords:** Glioma, TRPM7, FOSL1, NF-κB, Glioma stem cells, Signaling pathways

## Abstract

Glioblastoma multiforme (GBM) is the most common and malignant primary brain tumor; GBM’s inevitable recurrence suggests that glioblastoma stem cells (GSC) allow these tumors to persist. Our previous work showed that FOSL1, transactivated by the STAT3 gene, functions as a tumorigenic gene in glioma pathogenesis and acts as a diagnostic marker and potential drug target in glioma patients. Accumulating evidence shows that STAT3 and NF-κB cooperate to promote the development and progression of various cancers. The link between STAT3 and NF-κB suggests that NF-κB can also transcriptionally regulate FOSL1 and contribute to gliomagenesis. To investigate downstream molecules of FOSL1, we analyzed the transcriptome after overexpressing FOSL1 in a PDX-L14 line characterized by deficient FOSL1 expression. We then conducted immunohistochemical staining for FOSL1 and NF-κB p65 using rabbit polyclonal anti-FOSL1 and NF-κB p65 in glioma tissue microarrays (TMA) derived from 141 glioma patients and 15 healthy individuals. Next, mutants of the human FOSL1 promoter, featuring mutations in essential binding sites for NF-κB were generated using a Q5 site-directed mutagenesis kit. Subsequently, we examined luciferase activity in glioma cells and compared it to the wild-type FOSL1 promoter. Then, we explored the mutual regulation between NF-κB signaling and FOSL1 by modulating the expression of NF-κB or FOSL1. Subsequently, we assessed the activity of FOSL1 and NF-κB. To understand the role of FOSL1 in cell growth and stemness, we conducted a CCK-8 assay and cell cycle analysis, assessing apoptosis and GSC markers, ALDH1, and CD133 under varying FOSL1 expression conditions. Transcriptome analyses of downstream molecules of FOSL1 show that NF-κB signaling pathway is regulated by FOSL1. NF-κB p65 protein expression correlates to the expression of FOSL1 in glioma patients, and both are associated with glioma grades. NF-κB is a crucial transcription factor activating the FOSL1 promoter in glioma cells. Mutual regulation between NF-κB and FOSL1 contributes to glioma tumorigenesis and stemness through promoting G1/S transition and inhibiting apoptosis. Therefore, the FOSL1 molecular pathway is functionally connected to NF-κB activation, enhances stemness, and is indicative that FOSL1 may potentially be a novel GBM drug target.

**Supplementary Information:**

The online version contains supplementary material available at 10.1007/s00018-024-05293-1.

## Significance

The interplay between NF-κB and FOSL1 plays a crucial role in glioma tumorigenesis and stemness. FOSL1 promotes G1/S transition, inhibits apoptosis, and emerges as a promising novel drug target for GBM patients.

**Supplementary Information:**

The online version contains supplementary material available at 10.1007/s00018-024-05293-1.

## Introduction

Glioblastoma multiforme (GBM) is the most common and malignant primary brain tumor [1]. Patients with GBM have dismal prognosis despite rigorous therapy such as maximum surgical resections, radiation, and chemotherapy, with a median survival of ∼ 15 months [2]. Despite the deluge of research purporting to discover targets for GBM therapy, few have effectively translated into clinical care. Therefore, achieving a better understanding of the underlying molecular mechanisms that drive glioblastoma stem cell (GSC) maintenance, plasticity, and resistance will strengthen our capability to identify and develop novel and effective molecular-targeted therapies against GBM by selectively targeting and eliminating these tumor-initiating and -propagating populations.

Our earlier studies uncovered a pivotal event in glioma development- the activation of FOSL1, an integral member of the AP-1 transcription factor family, by the STAT3 gene. Specifically, our previous research highlighted how STAT3 triggers FOSL1 transcription by binding to the two GAS elements within the FOSL1 promoter, thereby enhancing glioma stemness [3]. FOSL1’s role as an oncogene in glioma progression underscores its promise as both a diagnostic marker and a feasible target for pharmacological intervention in glioma patients [3, 4]. We also demonstrated that deacetylation of FOSL1 at the Lys-116 residue located within its DNA binding domain led to an increase in FOSL1 transcriptional activity [3].

The Nucleofactor κB transcription factor (NF-*κ*B) family members, including five subunits, RelA (p65), RelB, c-Rel, p50, and p52, and five proteins with inhibitory activities, IκBα, IκBβ, IκBε, p105, and p100 [5]. NF-κB subunits interact with each other to form hetero- or homodimers. NF-κB plays a critical role in skin [6], ovarian [7], breast [8], cervical [9], colon, liver cancer [10], and GBM [11] through multiple mechanisms. NF-κB signaling induces the epithelial mesenchymal transition (EMT), assists in the acquisition of cancer stem cell (CSC) properties, and the self-renewal of CSC [8], contributing to neoplastic transformation and tumorigenesis [6].

There is mounting evidence indicating the collaborative role of STAT3 and NF-κB in advancing the onset and progression of diverse cancers [12–14]. Specifically, genes like FOSL1, housing both STAT3 and NF-κB binding sites, could be jointly regulated by these transcription factors [15, 16]. This synergy prompts exploration into whether or not NF-κB can orchestrate FOSL1’s transcription and potential bidirectional regulation between NF-κB and FOSL1, thereby influencing gliomagenesis. In this study, we will delineate the interaction of NF**-**κB, an evolutionarily conserved group of molecules, with FOSL1 in promoting glioma tumorigenesis and glioma stemness.

## Materials and methods

### Antibodies and reagents

The following primary antibodies were used in the present study: Rabbit polyclonal anti-TRPM7 (cat. no. ab23455) was purchased from Abcam. Rabbit polyclonal anti-FOSL1 antibody (cat. no. SAB2108461) for Immunohistochemistry (IHC) staining, rabbit polyclonal anti-β-actin antibody (cat. no. A3854) and mouse monoclonal anti-FLAG M2 antibody (cat. no. F1804) were purchased from Sigma-Aldrich. Rabbit polyclonal PE anti-human CD133 antibody (cat no. 372,804) and PE mouse IgG1, κ isotype ctrl (FC) antibody were purchased from BioLegend. Rabbit polyclonal anti-aldehyde dehydrogenase 1 antibody (ALDHA1; cat no. GTX123973) was purchased from GeneTex, Inc. Mouse monoclonal anti-FOSL1 antibody (cat. no. sc-283,107, for Western blotting), mouse monoclonal anti-NF-κB p65 antibody (cat. no. sc-8008), mouse monoclonal anti-p53 antibody (cat. no. sc-7974), mouse monoclonal anti-PTEN antibody (cat. no. sc-126), and mouse IKKβ monoclonal antibody (cat. no. sc-8014) were purchased from Santa Cruz Biotechnology. Rabbit monoclonal Phospho-NF-κB p65 (Ser536, cat. no.3033) antibody, mouse monoclonal IκBα (cat. no.4814 S), and phosphorylated IKKβ (Ser176/Ser180, cat. no. 2697) antibody were purchased from Cell Signaling. Phosphorylated IκBα antibody (Ser32/Ser36, cat. no. MA515224) and secondary rabbit Alexa 568 antibody (cat. no. A11011) were purchased from Life Technologies. All secondary antibodies (goat anti-rabbit, peroxidase-conjugated, cat. no. AP132P; and goat anti-mouse antibody, peroxidase-conjugated, cat. no. AP124P) used for Western blotting were purchased from Calbiochem; Merck KGaA.

### Plasmids and siRNA

Control scrambled siRNA (On-TARGETplus Non-targeting siRNA, cat. no. D-001810-01-05) and ON-TARGETplus SMARTpool siRNA targeting Human RELA NF-κB p65 (Cat. no. L-003533-00-0005), were purchased from Dharmacon (Lafayette, CO). FOSL1 human GFP-tagged ORF clone (RG202104) and control vector (PS100010) as well as FOSL1-Myc-DDK (FLAG)-tagged ORF (RC202104) and control vector PS100001 were purchased from Origene. pCMV4-HA (cat. no. 27,553) and pCMV4-p65 (cat. no. 21,966) were purchased from Addgene.

*Construction of site-specific mutants of the FOSL1 promoter at the putative NF-κB transcription factor binding sites and Luciferase Reporter Assays.* The human FOSL1 Luc has been generated as described in our previous publication [3]. The site-specific mutants that disrupted various NF-κB binding sites (mutant M1: -892 to -883, mutant M2: -349 to -342, mutant M3: -309 to -299, mutant M4: -164 to -155, mutant M5: -78 to -67, and the mutant with mutations in all five NF-κB binding sites M1-5) were created by a PCR-based approach using Q5 site-directed mutagenesis kit (NEB, cat. no. E0554S) as we described before [3]. We also generated a double mutant with mutations in both of the two STAT3 binding sites [3] and five NF-κB binding sites within the FOSL1 promoter (referred to as the double mutant). The primers used to make the mutations in the NF-κB binding sites are shown in Table 1. The primers utilized for inducing mutations in the STAT3 binding sites are described as previously [3]. For the reporter assays, 5 × 10^4^ cells were seeded in 24-well plates and transfected with either wild type (FOSL1 Luc) or a variety of mutants of FOSL1, along with NF-κB p65 construct, or siRNA NF-κB p65 (siNF-κB p65/siP65) into glioma cells for 24–72 h as needed. The protein lysates were made using a Dual-Luciferase Reporter (DLR) Assay system (Promega, cat. no. E1910). Firefly and *Renilla* luciferase activities were then measured using a DLR Assay system. The firefly luminescence was normalized to *Renilla* luminescence as an internal control for transfection efficiency. Experiments were performed three times as we described before [3].

**Table 1:** List of primers used in the study

Primer set	Forward 5’-3’	Reverse 5’-3’
FOSL1	CTCCAGGGGTACGTCGAAG	TCAGTTCCTTCCTCCGGTTC
P50 P65	GCAGCACTACTTCTTGACCACC ATCCCATCTTTGACAATCGTGC	TCTGCTCCTGAGCATTGACGTC CTGGTCCCGTGAAATACACCTC-
GAPDH	GAAGGTGAAGGTCGGAGTC	GAAGATGGTGATGGGATTTC
hFOSL1 Luc mut 1- M1	CAGCCTCAATTTCTCCATCTGTAAAACAGAGA	GGAGATGCTGAAATGAATTGCTTGAAGTCGCTA
hFOSL1 Luc mut 2-M2	TATGCTCGAGGCCTTAAAAC	TGAAAAGTGAATAAATGACA
hFOSL1 Luc mut 3-M3	ACTTCACGAGGCTTTGGGTG	GAGGTCTTTCCACTGGCCTT
hFOSL1 Luc mut 4-M4	AGGAAAACCCGGGGCTCCA	GCGGACTGACGCACCTGCCA
hFOSL1 Luc mut 5-M5	CGGAGAACGCTGAGCCGGGCC	GAGGCTGCGGGCCCGCCCCC
hFOSL1 ChIP for mut 1	CTGTGACTTGGCAACCTGTG	TGAGTGAATGTGAGGCAGGT
hFOSL1 ChIP for mut 3	CCTTAAAACAAGGCCAGTGGA	CCGTTTCTGCTCCCACAAAA

### Cell culture

Human glioblastoma cell lines: A172 (RRID: CVCL_0131) and U87MG (HTB-14), a glioblastoma of unknown origin (RRID: CVCL_0022), were obtained from American Type Culture Collection (ATCC). All cells were cultured in Dulbecco’s modified Eagle’s medium (DMEM) supplemented with 10% fetal bovine serum (FBS; both from Thermo Fisher Scientific, Inc.), 50 units/ml penicillin, and 50 µg/ml streptomycin at 37˚C. The PDX-L14 line, a patient-derived xenoline (PDX), was generated by subcutaneously implanting PDX-tumor tissue cubes into the flanks of male or female 6–8 weeks old nude mice, following the methodology outlined in our previous publication [3]. Subsequently, a single-cell suspension was obtained, and the cells were then grown in DMEM/F-12 media plus 10% FBS, 50 units/ml penicillin, and 50 µg/ml streptomycin for future use. All experiments were performed with mycoplasma-free cells.

### Transfection of siRNA and DNA constructs

When glioma cells reached approximately 50–75% confluency, specific siRNAs and their corresponding controls at a final concentration of 100 nM were transfected using Lipofectamine RNAiMAX reagent in serum-free OptiMEM-1 medium (Invitrogen, Carlsbad, CA), following the manufacturer’s instruction. For transfection of FOSL1 tagged with GFP or Myc-DDK, along with corresponding controls, various glioma cells at 50–75% confluency were transfected using lipofectamine 3000 transfection reagent (Invitrogen, Carlsbad, CA), according to the manufacturer’s instructions. Assessment of target knockdown or overexpression was performed 48–72 h post-transfection using Western blotting or immunofluorescence imaging, as required. All studies were done in triplicates. To assess the transfection efficiency, cells transfected with the GFP-tagged vector were either observed under an immunofluorescence phase-contrast microscope to visualize the GFP-tagged cells or assessed using flow cytometry.

### The cell counting assay (CCK-8)

The glioma cells were seeded at a density of 1 × 10^4^ cells in 100 µl of medium per well into 96-well plates. They were subsequently transfected with the appropriate amount of Myc-DDK-tagged FOSL1 and corresponding controls using Lipofectamine reagents for the specified duration. Following transfection, CCK-8 solution was added to the cells, and they were then incubated for 3 h. Subsequently, the absorbance of the solution was measured at 460 nm using the CytoFluorTM 2300 plate reader. Cell viability was determined by comparing the absorbance of the treated cells to control cells.

### Flow cytometry

To evaluate CD133 expression by flow cytometry, cells were harvested, washed with Cell Staining Buffer (cat. no. 420,201; Biolegend, Inc.), and then incubated with PE anti-human CD133 antibody (1:200; cat. no. 372,803; Biolegend, Inc.) for 15–20 min on ice in the dark. Cells were then washed and suspended in Cell Staining Buffer (at room temperature for 5 min) for analysis. The data acquired on the Guava easyCyte 8HT Base System were analyzed using the Flowjo software. ALDH1 enzymatic activity was assessed using an Aldefluor kit (cat. no. 01700; STEMCELL Technologies Inc.) according to the manufacturer’s instructions. Cells suspended in the Aldefluor assay buffer were incubated with ALDH enzyme substrate, BODIPY-aminoacetaldehyde (BAAA; 1:200), for 30–60 min at 37˚C. As a control for baseline fluorescence, cells were also treated for 30–60 min at 37˚C with the ALDH inhibitor, diethylaminobenzaldehyde (DEAB at 1:100 dilution). Fluorescence was detected using the Guava easyCyte 8HT Base System and analyzed using the Flowjo software. Statistical significance was determined by the paired Student’s t-test or one-way ANOVA test.

Cell cycle analysis and apoptosis assay: 1 × 10^6^ cells were harvested, fixed in ice-cold 70% ethanol, and resuspended in PBS for 1 min. After centrifuge at 450 x g for 5 min with the brake on low at room temperature, the cells were resuspended in 200 µl Guava Cell Cycle Reagent (cat no. 4500 − 0220, Luminex, Austin, TX) and incubated at room temperature for 30 min while shielded from the light. All samples were transferred to 96-well microplate plates with round bottoms and analyzed on a Guava easyCyte 8HT Base System. The percentage of cells in G0/G1, S, and G2/M phases was determined from the DNA content using guavaSoft 3.1.1. The apoptotic glioma cells were detected by flow cytometry using Annexin V-PE and 7-AAD. The staining procedure was conducted with a Guava Nexin Reagent kit (cat no. 4500 − 0455, Luminex) according to the manufacturer’s protocol. Briefly, after desired treatments, cells were collected and resuspended in 100 µl of 1% FBS (Cell concentration should be between 2 × 10^5^ and 1 × 10^6^ cell/ml) followed by incubation with 100 µl of Guava Nexin Reagent for 20 min at room temperature in the dark. The samples were then acquired on a Guava easyCyte 8HT Base System (Luminex) to detect apoptotic cells. Data were analyzed using the built-in software designed for analyzing apoptosis.

### Quantitative real-time RT-PCR (qRT-PCR)

Total RNA isolation, cDNA synthesis, and PCR amplification were performed as we previously described [17]. Total RNA was isolated from cells using a RNeasy Kit (Qiagen, Valencia, CA) and quantified using the Nanodrop N-1000 by Agilent Biosystems (Santa Clara, CA). Purified total RNA (0.75 µg) was reverse transcribed using the iScript cDNA Synthesis Kit (Bio-Rad Laboratories, Inc, Hercules, CA) according to manufacturer’s protocol. Reverse transcription was performed using random hexamers at 25 °C for 5 min, 42 °C for 30 min, and 85 °C for 5 min. After diluting ten times, the cDNA was then amplified using iQ SYBR Green Supermix (Bio-Rad Laboratories, Inc.) according to manufacturer’s protocol under the following conditions: activation of the Taq DNA polymerase at 95 °C for 3 min, 40 cycles at 95 °C for 10 s (denaturation), and 61 °C for 45 s (combined annealing and extension). The quantitative gene analysis utilized the CFX Connect Real-Time PCR Detection System. Each condition was conducted in biological triplicates, and each biological replicate was amplified in technical triplicates. Relative expression for each gene was evaluated using the 2^−ΔΔCt^ Livak method, and GAPDH was used as the reference gene [17]. The primers for FOSL1, p50, p65, and GAPDH were listed in Table 1.

### Western blotting

Cells were lysed with lysis buffer of M-PER™ Mammalian Protein Extraction Reagent (Thermo Fisher Scientific cat. no. 78,501) supplemented with Halt™ Protease and Phosphatase Inhibitor Single-Use Cocktail (100x) (Thermo Fisher Scientific cat. no. 78,442). SDS/PAGE separated samples and separated proteins were transferred to nitrocellulose membranes and identified by immunoblotting. Primary antibodies were obtained from commercial sources and were diluted to a ratio of 1:500 or 1:1000 according to manufacturer’s instruction. Blots were developed with Supersignal Pico or Femto substrate (Pierce). Densitometric analysis of the bands was performed with the ImageQuant program (Bio-Rad).

*Tissue microarray (TMA).* Glioma tissue arrays were purchased from BioCoreUSA Corporation (https://biocoreusa.com/default.aspx) and US Biomax, Inc. (https://www.biomax.us/). Biopsy features included age, sex, organ or anatomic site involved grading, and pathological diagnosis (H&E‑stained sections). Slides from BioCoreUSA (product no. GL1001b) contained 75 cases of glioma: grade II, *n* = 51 (astrocytoma, *n* = 47; oligodendroglioma, *n* = 2; oligoastrocytoma, *n* = 2); grade III, *n* = 12 (anaplastic astrocytoma); grade IV, *n* = 12 (glioblastoma), and 10 cases of normal brain tissues. Slides from Biomax (product no. GL803c) contained 68 cases of glioma: grade II, *n* = 27 (astrocytoma, *n* = 14; oligoastrocytoma, *n* = 13), grade III, *n* = 4 (astrocytoma); grade IV, *n* = 37 (glioblastoma, *n* = 31; pleomorphic glioblastoma, *n* = 6), and 5 cases of normal brain tissues.

### Immunohistochemistry (IHC)

IHC staining was performed on 5-µm thick microarray slides. The slides were baked at 55–60 °C for 30 to 60 min and pretreated in citrate buffer of pH 6 for 10 min at 100˚C. The slides were then incubated with different primary antibodies diluted at 1:100 at 4˚C overnight. The secondary antibody was diluted at 1:200 and incubated at room temperature for 60 min. A streptavidin-biotin unlabeled immunoperoxidase technique (ABC-Elite; cat. no. PK-6101, Vector Laboratories, Inc.) with diaminobenzidine (DAB) [DAB Substrate Kit, Peroxidase (HRP), cat. no. SK-4100, Vector Laboratories, Inc.] was used as a chromogen. Mayer’s hematoxylin was used for nuclear counterstaining for 2 min. The slides were then visualized under the light microscope.

*HSORE determination.* The staining intensity of cells in TMA was evaluated as negative or positive in three different bright fields (≥ 100 cells/field). The semi-quantitative HSCORE was calculated for different antigens such as p65 and FOSL1, using the following equation: HSCORE = Ʃpi (i + 1), where ‘i’ is the intensity with a value of 0, 1, 2, or 3 (negative, weak, moderate, or strong, respectively), and ‘pi’ is the percentage of stained cells for each intensity [4, 18, 19]. Immunohistochemically stained slides were blindly reviewed and scored by two independent investigators.

### ChIP-qPCR analysis

The chromatin immunoprecipitation (ChIP) coupled with quantitative PCR (qPCR) assay was performed using the Pierce Agarose ChIP Kit (Thermo Scientific, cat. no. 26,156). The glioma cells were crosslinked with 1% formaldehyde and inactivated by 125 mM glycine. Samples then undergone Micrococcal Nuclease digestion (MNase Digestion) generating chromatin fragments (0.2 ∼ 1 kb), which were then incubated with 5 µg of monoclonal anti-phosphorylated NF-κB p65 or normal mouse IgG (negative control) on a rocker overnight at 4 °C. Then, protein A/G agarose beads were added, and the chromatin was incubated for 1 h at room temperature. Antibody-bound protein/DNA complexes were eluted, reverse-crosslinked by incubation with proteinase K at 65 °C for 40 min and subjected to qPCR. An aliquot of chromatin that was not incubated with an antibody was used as the input control sample (10% total input sample). qPCR was performed using primers designed to surround the − 892 to -883 (M1) and − 309 to -299 (M3) binding sites of the FOSL1 promoter. The primer sequences for each site (M1 and M3) were listed in Table 1. Quantitative PCR was performed using Bio-Rad SYBR QPCR Master Mix (Bio-Rad, cat. no. 1,708,882) with the CFX Connect Real-Time PCR Detection System (Bio-Rad). Data were analyzed using the percent input method and using normal IgG as a negative control as shown in the reference [20].

### Nuclear extraction and NF-κB p65 transcription factor assay

Nuclear and cytoplasmic fractionation of glioma cells was carried out according to manufacturer instructions (Thermo Scientific cat. no. 78,833). Briefly, cells were collected with a cell scraper and pelleted by centrifugation. Pellets were re-suspended in the pre-extraction buffer. After centrifugation, the supernatant containing cytoplasmic fraction was frozen and stored. The pellet was again centrifuged and re-suspended in a nuclear extraction buffer to generate the nuclear fraction. The nuclear fraction was employed for NF-κB p65 transcription assays (NF-κB p65 activity) utilizing a p65 binding ELISA kit following the manufacturer’s instructions (Abcam, cat. no. ab133112). The resulting values were normalized based on microgram protein levels determined through protein estimation.

### RNA sequencing

PDX-L14 cells were transfected with GFP-tagged FOSL1 and corresponding controls. At 72 h, the total RNA of each sample was extracted from the cells using the TRIzol reagent. RNA seq was conducted by Novogene (Sacramento, CA) using 3 replicates of samples for each group. Differentially expressed genes (DEGs) in the FOSL1 overexpression group was defined with values of *p* < 0.05 and fold change ≥ 2, when compared with the vector control group using DEseq2. Gene enrichment analysis was conducted using gene set enrichment analysis (GSEA) software [21, 22] to investigate the signaling pathways regulated by FOSL1.

### Statistical analysis

The results obtained in the present study are expressed as the mean ± SD of at least 3 independent experiments conducted in triplicates. GraphPad Prism 9 (GraphPad Software, Inc.) was used for statistical analysis. Paired Student’s t-test or one-way ANOVA followed by Holm-Sidak post hoc tests were performed for data analysis, and *p* < 0.05 was considered to indicate a statistically significant difference. Pearson correlation analysis was performed to determine possible correlations between the level of FOSL1 and other parameters such as TRPM7 and NF-κB activities.

## Results

### Transcriptome analyses of downstream molecules of FOSL1 indicate that NF-κB signaling pathways are regulated by FOSL1

We have reported that FOSL1 promotes glioma proliferation, invasion and enhances glioma stemness [3, 4]. We observed that the proneural (PN) subtype PDX-L14/X456 exhibited a notably low level of FOSL1 in comparison to the other glioma cell lines (refer to Fig. 4D in reference [4]).

**Fig. 1:**
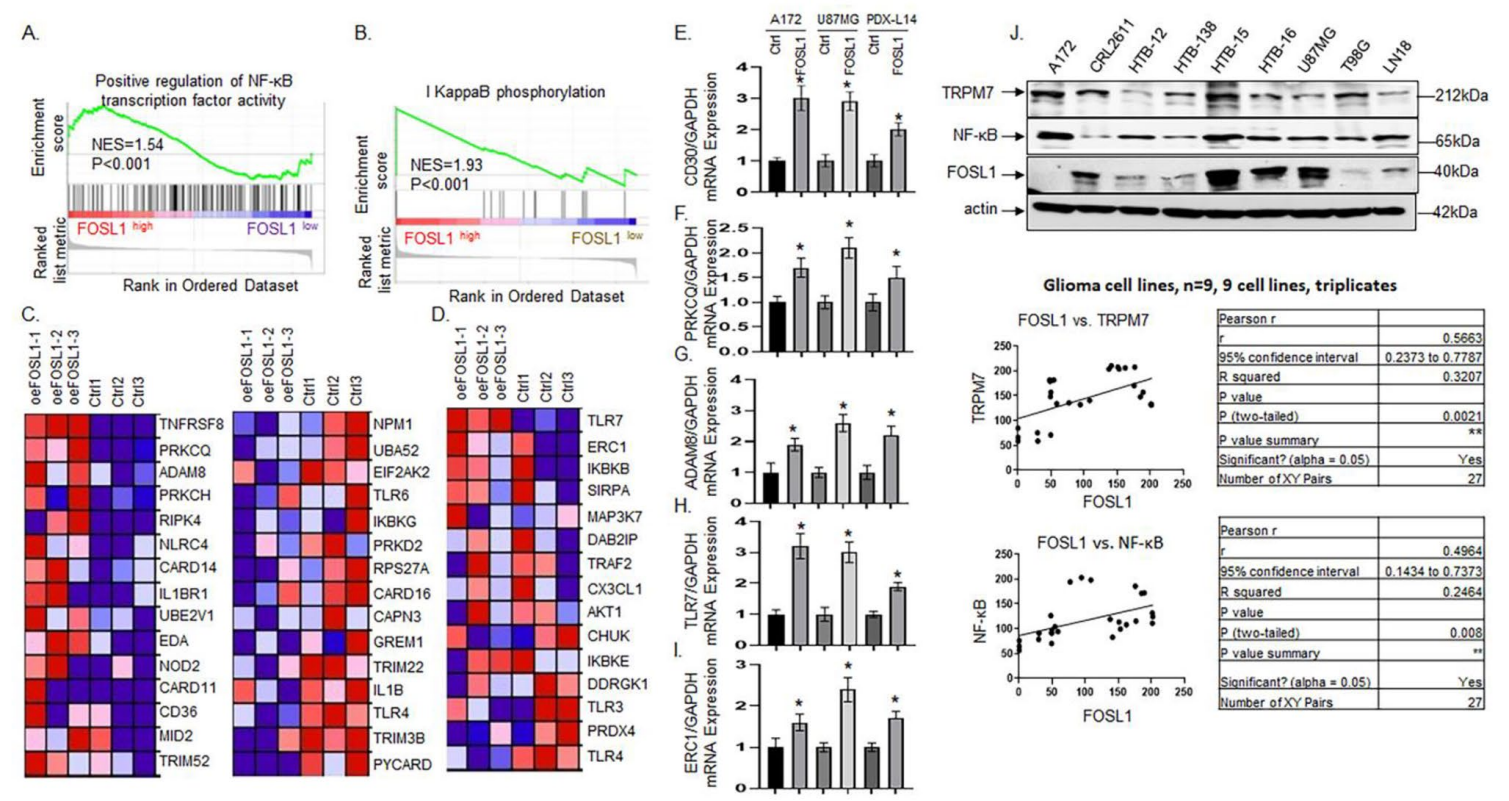
GSEA enrichment of pathways associated with the regulation of NF-κB signaling. (**A**) The GSEA enrichment of positive regulation of NF-κB transcription factor activity. (**B**) The GSEA enrichment of IκB phosphorylation. (**C, D**) Heat maps showing the top 15 genes and last 15 genes corresponding to the positive regulation of NF-κB signaling pathways in panel A (**C**) and all 15 genes involved in κB phosphorylation signaling pathways in panel B (**D**). (**E-I**) qPCR Validation of the top 3 differentially expressed genes in panel C (**E**: CD30; **F**: PRKCQ; and **G**: ADAM8) and top 2 in panel D (**H**: TLR7; **I**: ERC1) in A172, U87MG, and PDX-L14 cells with overexpressed FOSL1. The endogenous FOSL1, TRPM7 and NF-κB protein expression levels were examined in glioma cell lines by Western blot (**J** upper panel) and the correlation between FOSL1 and TRPM7 (**J** middle panel), as well as between FOSL1 and NF-κB (**J** lower panel) was analyzed by Pearson’s correlation in GraphPad

FOSL1 is a key regulator of the transition from PN to mesenchymal (MES), which is associated with glioma progression [23, 24]. To identify the role of FOSL1 in GBM transition and progression, we transfected PDX-L14 cells (which have very low expression of FOSL1) with FOSL1 tagged with GFP to overexpress FOSL1, along with vector control by lipofectamine 3000, followed by RNA-seq and transcriptome analyses. GSEA analysis revealed that FOSL1 activates the positive regulation of the NF-κB activity (Fig. 1A) and IκB phosphorylation (Fig. 1B). The heatmap representing each signaling pathway was depicted in Fig. 1C [For clarity, only the top (left panel) and last 15 genes (right panel) are shown, and the remaining 97 genes are provided in Supplementary Table 1] and Fig. 1D, respectively. Subsequent qPCR validation confirmed a significant increase in mRNA levels of CD30 (Fig. 1E), PRKCQ (Fig. 1F), and ADAM8 (Fig. 1G) in glioma cell lines (A172, U87MG, and PDX-L14) overexpressing FOSL1, which are key regulators at the apex of the NF-κB signaling pathway. Additionally, TLR7 (Fig. 1H) and ERC1 (Fig. 1I), situated at the top of the IκB phosphorylation signaling pathway, also exhibited heighted expression compared to the vector control. These findings collectively suggest that FOSL1 plays a crucial role in regulating the NF-κB signaling pathway.

**Fig. 2:**
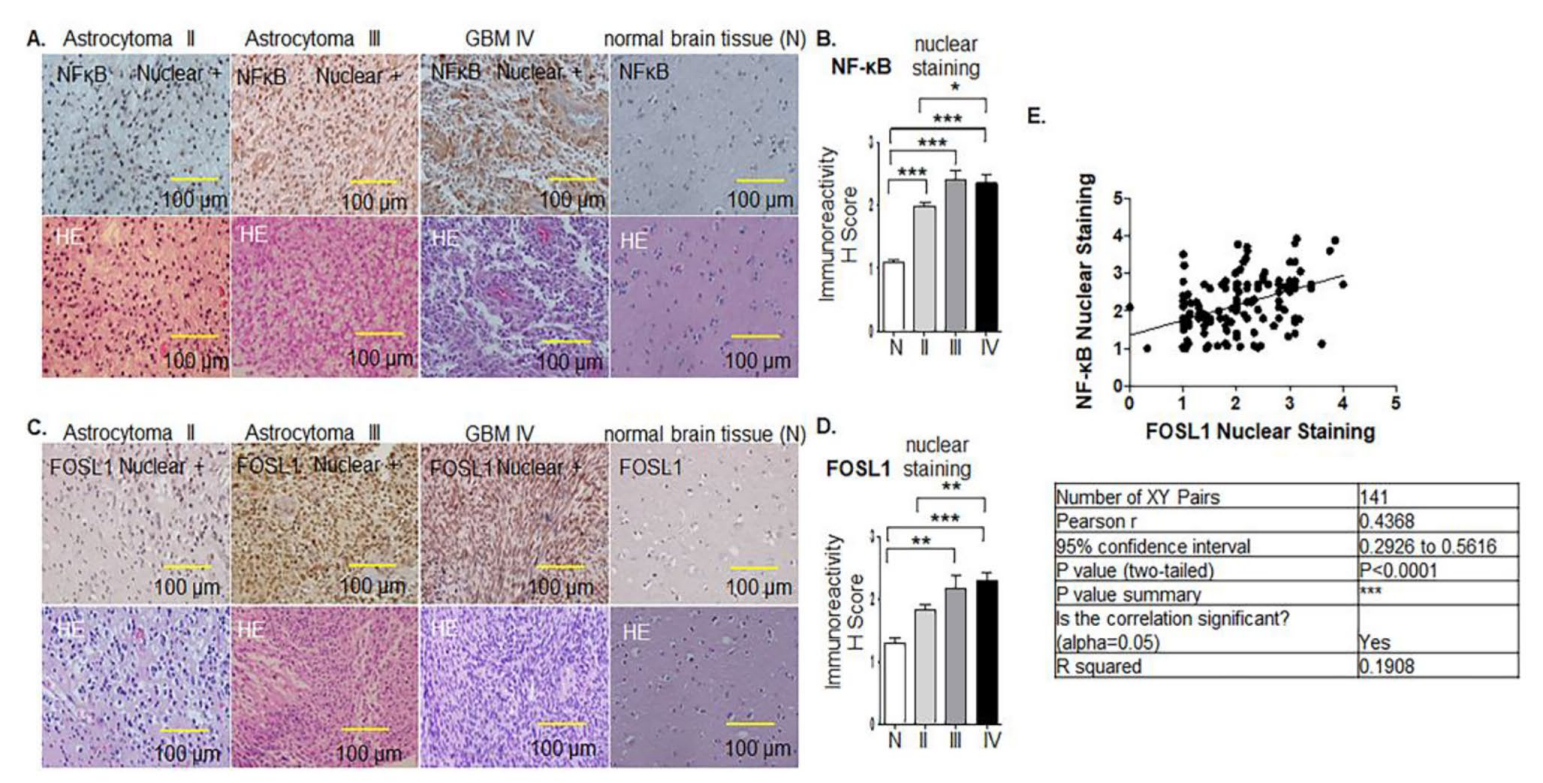
NF-κB (p65) protein expression correlates to the expression of FOSL1 in glioma patients. (**A**) IHC staining of NF-κB protein in brain tissues of astrocytoma II (left), astrocytoma III (middle left), GBM (middle right) of WHO grade patients, and normal brain tissues (right). (**B**) One-way ANOVA was used to analyze NF-κB expression between normal and glioma patients and among different grades of glioma. *p < 0.05, **p < 0.01, and *** *p* < 0.001. The HE images were positioned below each NF-κB image. (**C**) FOSL1 protein was expressed in grade II (left), astrocytoma III (middle left), grade IV GBM (middle) of WHO grade IV patients, and normal brain tissue (right). (**D**) Quantification of IHC results corresponding to (**C**), One-way ANOVA was used to analyze FOSL1 expression between normal and glioma patients and among different grades of glioma. *p < 0.05, **p < 0.01, and *** *p* < 0.001. (**E**) Pearson’s correlation curve revealed the positive correlation between the nuclear staining of FOSL1 and NF-κB

We have been extensively researching the role of the cation ion channel TRPM7 in glioma initiation and progression. Moreover, our investigation has revealed that TRPM7 regulates the non-coding RNAs miR-301a-3p and HOTAR and, further, positively regulates FOSL1 itself [4]. In addition to establishing the correlation between FOSL1 and NF-κB, we have also explored the relationship between FOSL1 and its upstream gene TRPM7. We have determined the FOSL1 protein’s expression in glioma cells. As shown in Fig. 1J, A172, CRL2611, HTB-12, HTB-138, HTB-15, HTB-16, U87MG, T98G, and LN18 cells express certain amount of FOSL1, with A172 and T98G having extremely lower levels of expression. We conducted an analysis of TRPM7 and NF-κB/p65 protein expression through Western blot (Fig. 1J) and subsequently performed densitometric analysis on triplicated samples of glioma cells using the ImageQuant program. Pearson’s correlation analysis revealed a positive correlation between FOSL1 and TRPM7 (Fig. 1J middle panel), as well as between FOSL1 and NF-κB/p65 (Fig. 1J lower panel). The latter correlation was further supported by immunohistochemistry (IHC) analysis conducted in glioma patients, as depicted in Fig. 2.

We also investigated whether GFP influences the localization and function of FOSL1. Firstly, we transfected FOSL1 tagged with GFP and the same vector tagged with Myc-DDK into glioma cells A172, U87MG, and PDX-L14. Subsequently, we examined FOSL1 localization using immunofluorescence. Our findings indicate that nuclear localization of FOSL1 was not affected by GFP, as observed through immunofluorescence staining, in comparison to cells transfected with Myc-DDK tag. These results are presented in Supplementary Fig. 1. Secondly, we transfected both GFP-tagged FOSL1 and Myc-DDK-tagged FOSL1 (note that both constructs use the same backbone vector) and subsequently performed qPCR to assess the expression of FOSL1 target genes and GSC markers CD133 and ALDH1. Our data demonstrate that GFP did not influence the expression of downstream genes of FOSL1, including CD133 and ALDH1, suggesting that GFP does not affect the function of FOSL1. These results are provided as Supplementary Fig. 2.

### NF-κB protein expression correlates to the expression of FOSL1 in glioma patients, and both are associated with different glioma grades

We assessed the protein expression of NF-κB/p65 (NF-κB or p65) through IHC using a glioma brain tissue microarray (TMA) compiled from glioma patients obtained from BioCoreUSA and US Biomax. NF-κB protein was mainly expressed in the nucleus. Little to no staining was found in normal brain tissues (Fig. 2A). Quantification of the IHC results showed that NF-κB’s positive nuclear staining was significantly higher in grade II astrocytoma (*n* = 76, *p* < 0.001, Fig. 2A left panel), grade III (*n* = 16, *p* < 0.001, middle left), and GBM (*n* = 49, *p* < 0.001, Fig. 2A middle right panel) compared to that of normal brain tissue (*n* = 15) (Fig. 2A right panel). Additionally, we found that NF-κB’s nuclear staining significantly increased in GBM compared to that in grade II (*p* < 0.05) (Fig. 2B).

Figure 2C showed the representative nuclear staining of FOSL1 in grade II astrocytoma (Fig. 2C left), astrocytoma III (middle left), and grade IV GBM (Fig. 2C middle right), as well as very weak nuclear staining in normal brain tissues (Fig. 2C right). Quantification of IHC results revealed that positive nuclear staining of FOSL1 was significantly higher in grade III (*n* = 16, *p* < 0.001) and grade IV GBM (*n* = 51, *p* < 0.001) compared with that of normal brain tissues (*n* = 15) (Fig. 2D). The expression of FOSL1 increased in grade II (*n* = 77) compared to that of normal individuals, although it did not reach statistical significance. The positive association between increased FOSL1 protein expression and glioma grades strongly implicated FOSL1 protein as a diagnostic marker and potential drug target for glioma patients.

Furthermore, Pearson’s correlation curve revealed the positive relationship between nuclear staining of FOSL1 and NF-κB (Fig. 2E). The positive correlation between increased NF-κB protein expression and glioma grade as well as its positive correlation with FOSL1 expression strongly indicates NF-κB’s potential as a prognostic marker in glioma patients.

### Transactivation of the FOSL1 promoter by NF-κB in glioma cells

**Fig. 3:**
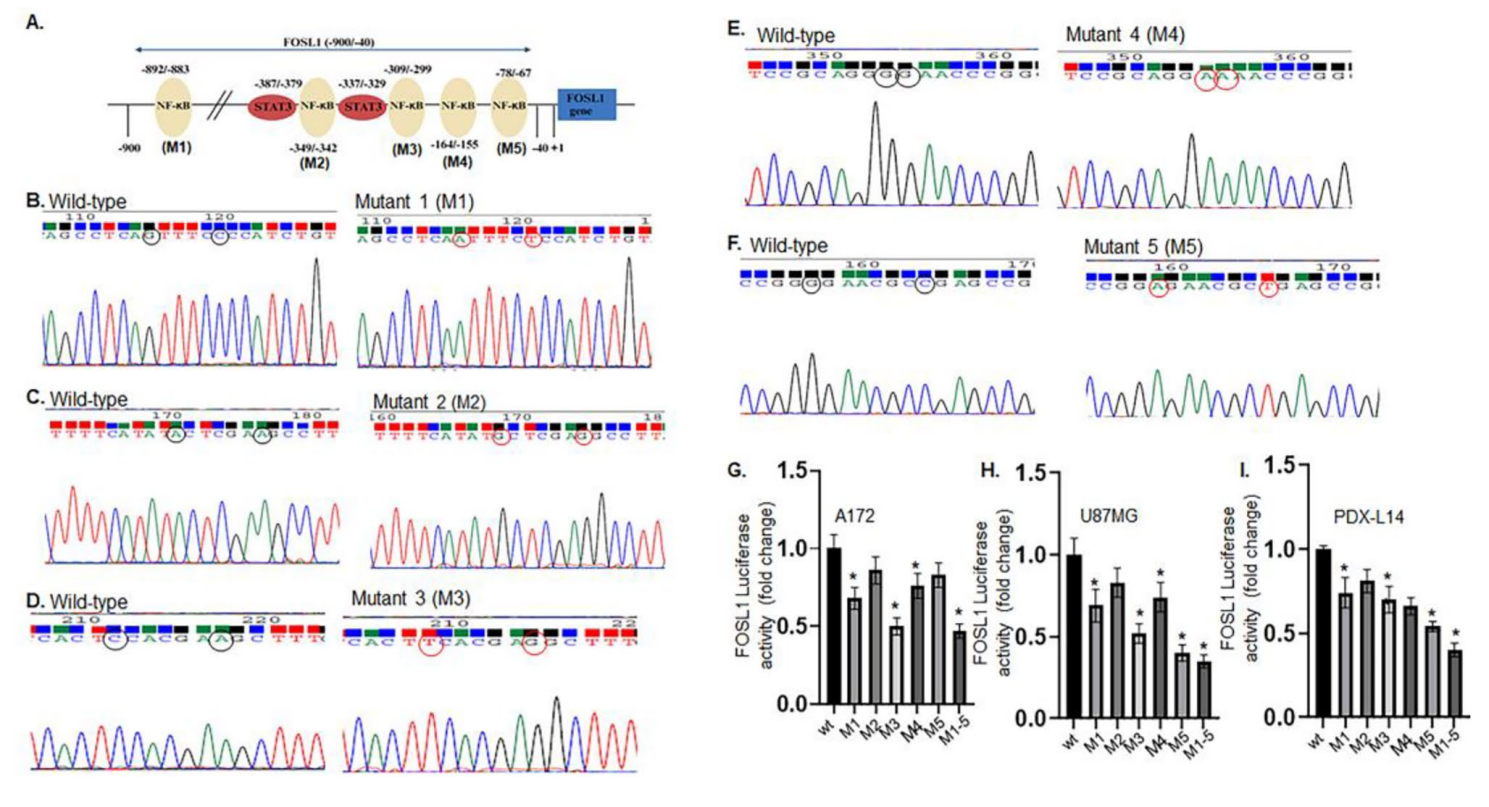
Transactivation of the FOSL1 promoter by NF-κB in glioma cells. (**A**) Transcription factor binding site prediction in FOSL1’s gene promoter by the Ensemble Genome Brower and PROMO program. (**B-G**) Site-specific mutation of the NF-κB binding sites of the FOSL1 promoter markedly reduced the promoter’s activity. The primers hybridizing to the most 5’ extent of the − 1000 FOSL1 promoter sequence were used to create the FOSL1 promoter and its five NF-κB mutants. These mutants were designated as M1 (**B**), M2 (**C**), M3 (**D**), M4 (**E**), and M5 (**F**). The left panel of each pair showed the wild-type sequence, while the right panel showed that NF-κB binding sites were eliminated by introducing a two-base change in each site. A combined mutant (M1-5) was also created in which all five NF-κB binding sites were mutated. (**G-I**) These constructs were transiently transfected into A172 (left, **G**), U87MG (middle, **H**), and PDX-L14 (right, **I**) cells. Following a 24-hour incubation period, cells were lysed and analyzed for luciferase activity

We located the FOSL1 promoter sequence through Ensembl Genome Browser, followed by a virtual laboratory PROMO program to identify putative NF-κB transcription factor binding sites. We found five predicted binding sites of NF-κB (Fig. 3) in FOSL1’s promoter. Therefore, we hypothesize that NF-κB induces FOSL1 transcriptional activation and contributes to gliomagenesis. To test our hypothesis and identify the potential roles for NF-κB’s binding sites on activating FOSL1 transcription, we used the promoter (wild-type FOSL1-luc or wtFOSL1-luc) we made previously [3] to generate five mutants in which a few critical bases in each NF-κB binding sites were mutated as we described before [3], M1 (-892 to -883, CAGTTTCCCC, Fig. 3B), M2 (-349 to -342, ATACTCGAA, Fig. 3C), M3 (-309 to -299, CTCCACGAAGC, Fig. 3D), M4 (-164 to -155, GGGGAACCCG, Fig. 3E), M5 (-78 to -67, GGGGAACGCCGA, Fig. 3F), and the construct in which all five NF-κB binding sites were mutated (M1-5). The promoter/reporter constructs were then transiently transfected into A172, U87MG, and PDX-L14 cells, and luciferase reporter assays were performed as described in our publication [3]. As shown in Fig. 3G to I, the M1, M3, M4, and M5 mutants induced varying degrees of reduction in FOSL1 promoter activity in all three cell lines. The presence of the combined mutant (M1-5) appears to exhibit synergistic effects, leading to a more substantial reduction in FOSL1 promoter activity, ranging from 53 to 65% across all three cell lines (Fig. 3G to I). Evidently, the presence of intact NF-κB binding sites is essential for maximal FOSL1 promoter activity.

We previously established that STAT3 regulates FOSL1 transcription [3]. To explore potential interplay between STAT3 and NF-κB, we generated a double mutant with mutations in both of the two STAT3 binding sites [3] and five NF-κB binding sites within the FOSL1 promoter (referred to as the double mutant, Supplementary Fig. 3A). Due to space limitations and for clarity and simplicity, only the sequence portion of the NF-κB binding site mutation (M2) and STAT3 binding site mutation were presented for the double mutant in Supplementary Fig. 3A. Subsequently, we transfected glioma cell lines A172, U87MG, and PDX-L14 with constructs containing FOSL1 wild type promoter (FOSL1 luc), as well as variants harboring mutations in the NF-κB combined binding site (M1-5), mutations in the STAT3 binding site (STAT3 mut), and the double mutant. Subsequently, we conducted luciferase assays. Our results revealed that while STAT3 mutation and NF-κB M1-5 mutant significantly reduced FOSL1 transactivation, the double mutant exhibited a greater inhibit of FOSL1 transcriptional activity compared to individual mutations in STAT3 and NF-κB (Supplementary Fig. 3B). This suggests an interplay and synergistic effect between STAT3 and NF-κB towards FOSL1 activity.

### NF-κB induces FOSL1 transcriptional activation in gliomagenesis

**Fig. 4:**
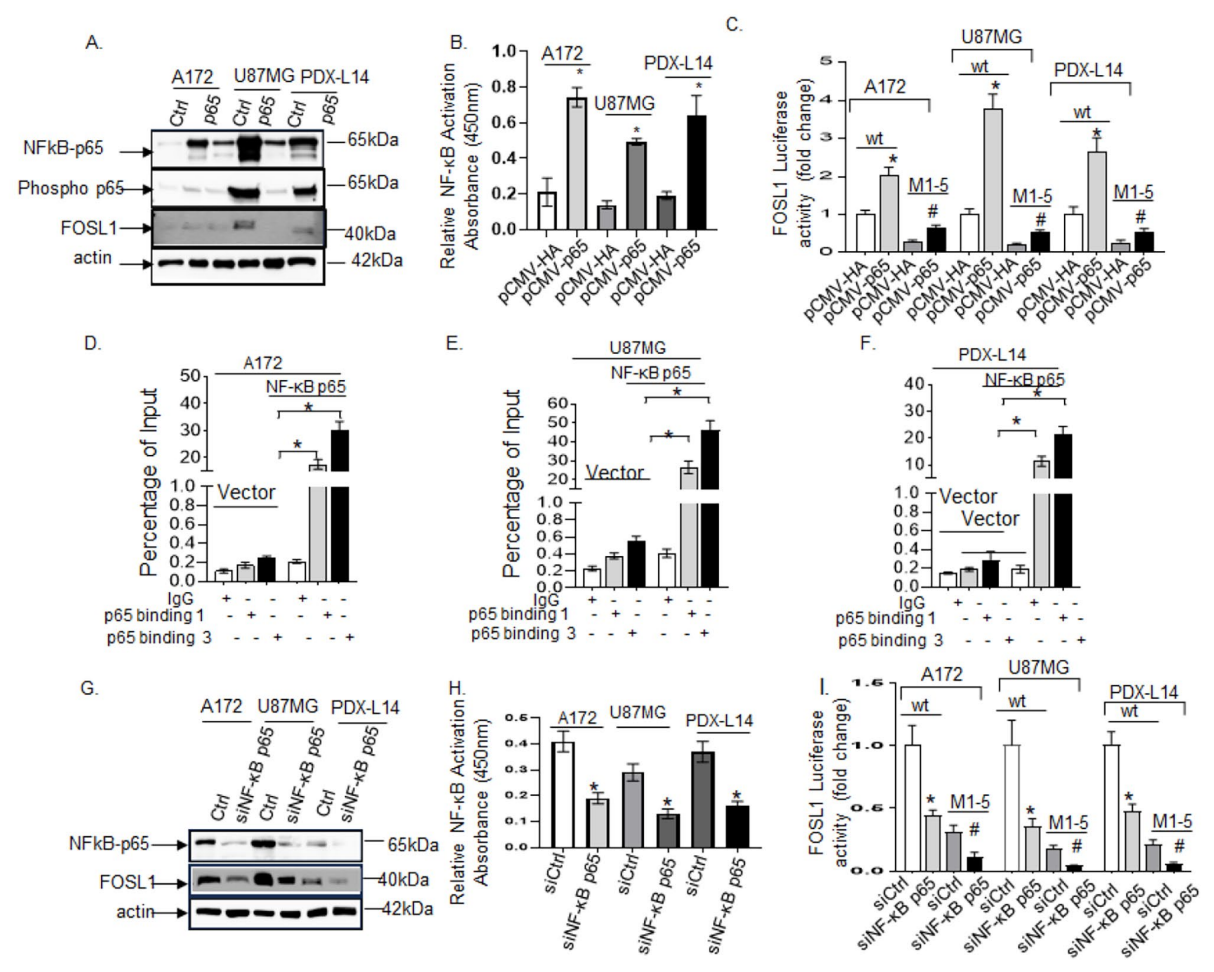
The positive role of NF-κB in transactivating the FOSL1 gene and amplifying its expression. (**A**) A172, U87MG, and PDX-L14 cells transfected with pCMV-p65 were assessed for total and phosphorylated NF-κB and FOSLl1 protein levels via Western blot. (**B**) Similar cells treated as in (**A**) underwent an ELISA to measure relative NF-κB activity in nuclear fraction lysates (* indicates *p* < 0.05 compared to the control using a Student’s t-test). (**C**) The pCMV p65 were transiently transfected into A172, U87MG, and PDX-L14 cells alongside wt-FOSL1 luc or mutant M1-5 constructs. Subsequently, luciferase activity was analyzed after a 24-hour incubation. (**D-F**) ChIP-qPCR analysis of direct binding to the FOSL1 promoter in A172 cells (**D**), U87MG cells (**E**), and PDX-L14 cells (**F**) transfected with pCMV-p65 and vector. ChIP-qPCR results were analyzed by evaluating the signal of enrichment of over noise normalized to input. DNA levels were normalized to relative inputs (*n* = 3 independent experiments; * indicates *p* < 0.05 among groups using one-way ANOVA). (**G**) Western blot analysis of NF-κB and FOSL1 protein levels in A172, U87MG, and PDX-L14 cells transfected with siNF-κB p65. (**H**) A similar treatment in (**G**), followed by an ELISA for relative NF-κB activity in nuclear fraction lysates (* indicates *p* < 0.05 compared to control using a Student’s t-test). (**I**) Transient transfection of siNF-κB p65 into A172, U87MG, and PDX-L14 cells alongside wt-FOSL1 luc or mutant M1-5 constructs, followed by analysis of luciferase activity as described in (**C**)

To further investigate the mechanism of NF-κB regulation of FOSL1, we transfected A172, U87MG and PDX-L14 with wild-type human NF-κB p65 pCMV4-p65 (pCMV-p65) and control vector pCMV4-HA (pCMV-HA). The transfection efficiency was first determined by Western blot. A172, U87MG, and PDX-L14, transfected with NF-κB p65 constructs, expressed high NF-κB p65 protein levels compared to that of the controls (Fig. 4A). We then examined the transfected glioma cells for NF-κB activity; We observed increased p65 (Ser536) phosphorylation in all three cell lines overexpressing NF-κB p65 (Fig. 4A); Furthermore, a NF-κB p65 binding ELISA for relative NF-κB activity on nuclear fraction lysates showed nuclear NF-κB p65 transcriptional activity was significantly increased in all three glioma cells overexpressing NF-κB p65 (Fig. 4B). Interestingly, the activation of phosphorylated NF-κB p65 led to a notable increase in the expression of FOSL1 (Fig. 4A), suggesting that FOSL1 functions downstream of NF-κB.

Mechanistically, to ascertain if NF-κB transactivates FOSL1, we transiently overexpressed NF-κB p65 in A172, U87MG, and PDX-L14 along with cotransfection of the wtFOSL1-luc and M1-5 mutant construct, followed by measuring the FOSL1 luc activity. The luc activity assays revealed notable findings illustrated in Fig. 4C. Specifically, there was a significant increase in FOSL1 transcriptional activity: a 2-fold, 3.7-fold, and 2.6-fold rise in A172, U87MG, and PDX-L14 cells respectively, upon overexpression of NF-κB p65. Conversely, a significant decrease in FOSL1 transcriptional activity was observed in the NF-κB binding site of mutant M1-5. The findings suggest that NF-κB plays a positive role in transactivating FOSL1 during gliomagenesis.

Furthermore, we investigate whether NF-κB p65 present in the nuclear lysate of glioma cells could bind to the FOSL1 promoter by performing ChIP-qPCR. To this end, we chose two NF-κB binding sites, M1 and M3. A172 (Fig. 4D), U87MG (Fig. 4E), and PDX-L14 cells (Fig. 4F) transfected with pCMV-p65 and vector pCMV-HA were crosslinked with 1% formaldehyde and underwent Micrococcal Nuclease digestion (MNase Digestion). The chromatin fragments generated (0.2 ∼ 1 kb) were then incubated with 5 µg of monoclonal anti-phosphorylated NF-κB p65 or normal rabbit IgG (negative control). Antibody-bound protein/DNA complexes were eluted and subjected to qPCR. An aliquot of chromatin that was not incubated with an antibody was used as the input control sample (10% total input sample). qPCR was performed using primers designed to surround the − 892 to -883 (M1) and − 309 to -299 (M3) binding sites of the FOSL1 promoter. The primer sequences for each site are listed in Table 1. Data were analyzed using the percent input method and using normal mouse IgG as negative controls. The ChIP experiments were performed in triplicates, and the results were presented together with the background signal and standard error. We made an intriguing discovery: in A172 cells (Fig. 4D; Table 2), the cp-immunoprecipitation of phospho-NF-κB p65 revealed a higher capture of FOSL1 promoter fragments (17.56% in binding 1 and 30.35% in binding 3) compared to the empty vector (0.21%). A parallel trend was evident in U87MG cells (Fig. 4E; Table 3), where a notable increase was observed (26.61% in binding 1 and 46.22% in binding 3) compared to the empty vector (0.41%). This trend persisted in PDX-L14 cells (Fig. 4F; Table 4) with similar findings (11.34% in binding 1 and 21.22% in binding 3 vs. 0.19% in the vector). These results demonstrated that NF-κB bound directly to the FOSL1 promoter region at the level of NF-κB putative binding sites and suggested that it potentially served as a FOSL1 transcription regulator.

**Table 2:** Calculation of percent input of A172

A172					
**Vector**					
		Step 1			Step 2
		*Adjusted input to 10%			Percent input
	Raw Ct	(Ct Inpu-3.32)		Triplicate average Ct	100*2^ (Adjusted input-Ct(IP)
Input (10%)	27.97	24.65	Adjusted input	24.65	
			Negative (IgG)	34.43	0.11
			pNF-κB p65 Antibody binding 1	33.86	0.17
			pNF-κB p65 Antibody binding 3	33.3	0.25
**pCMV-p65**					
		Step 1			Step 2
		*Adjusted input to 10%			Percent input
	Raw Ct	(Ct Inpu-3.32)		Triplicate average Ct	100*2^ (Adjusted input-Ct(IP)
Input (10%)	27.97	24.65	Adjusted input	24.65	
			Negative (IgG)	33.54	0.21
			pNF-κB p65 Antibody binding 1	27.16	17.56
			pNF-κB p65 Antibody binding 3	26.37	30.35

**Table 3:** Calculation of percent input of U87MG

U87MG					
**Vector**					
		Step 1			Step 2
		*Adjusted input to 10%			Percent input
	Raw Ct	(Ct Inpu-3.32)		Triplicate average Ct	100*2^ (Adjusted input-Ct(IP)
Input (10%)	29.31	25.99	Adjusted input	25.99	
			Negative (IgG)	34.77	0.23
			pNF-κB p65 Antibody binding 1	34.04	0.38
			pNF-κB p65 Antibody binding 3	33.48	0.55
**pCMV-p65**					
		Step 1			Step 2
		*Adjusted input to 10%			Percent input
	Raw Ct	(Ct Inpu-3.32)		Triplicate average Ct	100*2^ (Adjusted input-Ct(IP)
Input (10%)	29.31	25.99	Adjusted input	25.99	
			Negative (IgG)	33.92	0.41
			pNF-κB p65 Antibody binding 1	27.90	26.61
			pNF-κB p65 Antibody binding 3	27.10	46.22

**Table 4:** Calculation of percent input of PDX-L14

PDX-L14					
**Vector**					
		Step 1			Step 2
		*Adjusted input to 10%			Percent input
	Raw Ct	(Ct Inpu-3.32)		Triplicate average Ct	100*2^ (Adjusted input-Ct(IP)
Input (10%)	28.92	25.60	Adjusted input	25.60	
			Negative (IgG)	34.99	0.15
			pNF-κB p65 Antibody binding 1	34.68	0.19
			pNF-κB p65 Antibody binding 3	34.01	0.29
**pCMV-p65**					
		Step 1			Step 2
		*Adjusted input to 10%			Percent input
	Raw Ct	(Ct Inpu-3.32)		Triplicate average Ct	100*2^ (Adjusted input-Ct(IP)
Input (10%)	28.92	25.60	Adjusted input	25.60	
			Negative (IgG)	34.66	0.19
			pNF-κB p65 Antibody binding 1	28.74	11.34
			pNF-κB p65 Antibody binding 3	27.84	21.22

To further verify the biological function of NF-κB in activating FOSL1, we conducted transient transfection of siRNA NF-κB p65 (siNF-κB p65 or siP65) in A172, U87MG, and PDX-L14 glioma cells, along with corresponding controls. The efficiency of this transient transfection was initially assessed via Western blot analysis. As depicted in Fig. 4G, treatment with siNF-κB notably decreased the expression levels of NF-κB protein across all glioma cell lines. This reduction in NF-κB protein expression was further confirmed through a significant decrease in the nuclear NF-κB p65 transcriptional activity, as measured by a p65 binding ELISA (Fig. 4H). Interestingly, the reduced activity of NF-κB p65, achieved through knockdown, resulted in a significant decrease in FOSL1 protein expression (Fig. 4G) and a corresponding reduction in its transactivating effect on FOSL1, as measured by wtFOSL1-luc activity (Fig. 4I). Specifically, this led to a substantial decrease in FOSL1 transcriptional activity: a 56%, 75%, and 52% decrease in A172, U87MG, and PDX-L14 cells, respectively (Fig. 4I). Collectively, these findings support the positive role that NF-κB plays in transactivating the FOSL1 gene and enhancing its expression.

### FOSL1 positively regulates NF-κB and decreases p53 and PTEN protein expressions in glioma cell lines with wild type p53 (wtp53) and/or PTEN (wtPTEN)

**Fig. 5:**
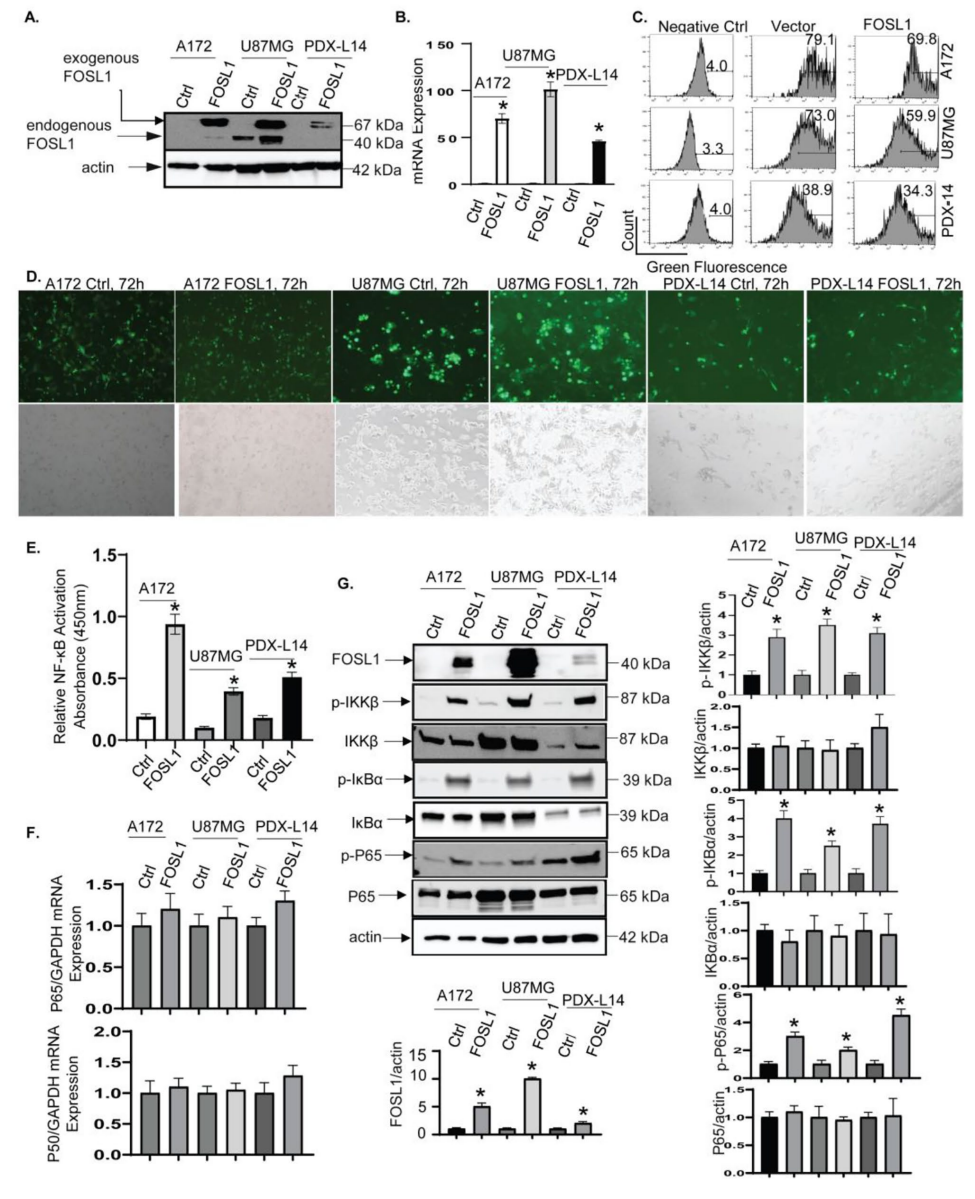
Overexpression of FOSL1 in GBM positively influences NF-κB. (**A-D**) A172, U87MG, and PDX-L14 cells were transfected with GFP-tagged human FOSL1 (FOSL1) and controls (Ctrl) followed by assaying protein expression of FOSL1 and GFP by Western blot (**A**), mRNA expression of FOS1 by qPCR (**B**), GFP positive cell by flow cytometry (**C**), and by immunofluorescence staining (**D**: A172-left, U87MG-middle, and PDX-L14-right). (**E**) The identical cellular samples, treated as detailed in (**A-D**), were subjected to ELISA analysis to quantify the relative NF-κB activity in nuclear fraction lysates (* denotes significance at *p* < 0.05 compared to control using a Student’s t-test). (**F**) Subsequent to the procedures outlined in (**E**), qPCR was conducted to evaluate the mRNA levels of NF-κB p65 and p50. (**G**) Following the protocols described in (**E**), Western blotting was employed to examine the levels of FOSL1 protein, along with phosphorylated (p-IKKβ) and total IKKβ, phosphorylated (p-IKBα) and total IKBα, and phosphorylated (p-P65) and total NF-κB p65. Densitometry analysis for Western blots was performed using the Image Quant program, with the corresponding results positioned below or adjacent to each image. The bar graphs represent the mean ± S.D. of three independent experiments

Recently HDAC2 has been identified as one of the top binding partners of FOSL1 [25]. Transformation of cells could be caused by elevated HDAC2 via inactivation of p53 [26]. HDAC2 and other HDACs might also integrate the crosstalk of p53 with other transcription factors, such as NF-κB [27, 28], STAT1 [29, 30], and STAT3 [31]. High HDAC2 levels can turn off pro-apoptotic functions of p53 [32]. HDAC2 also interferes with PTEN function [33, 34]. Hence, we investigated the responsiveness of the p53 and PTEN pathways to FOSL1 by evaluating the levels of p53 or PTEN proteins in glioma cell lines known for their functional status of p53 (wtp53), such as A172, U87MG, and for the functional PTEN status (wtPTEN) like PDX-L14 cells. Additionally, leveraging RNA-seq data (Fig. 1), we validated the impact of FOSL1 on NF-κB regulation. To address these objectives, we transfected the three glioma cell lines with FOSL1-GFP expressing constructs to augment FOSL1 expression levels, alongside control transfections. Subsequently, we assessed p53, PTEN and NF-κB activities. The effects of FOSL1 on the expression of p53 and PTEN were then detected by Western blot. The high transfection efficiency was revealed by Western blot (Fig. 5A), qPCR (Fig. 5B), flow cytometry (Fig. 5C), and immunofluorescence staining for GFP (Fig. 5D), respectively in all three glioma cell lines. The heightened expression of FOSL1 resulted in the increased activity of NF-κB p65, as evidenced by a p65 binding ELISA (Fig. 5E). We then sought to elucidate the mechanism through which FOSL1 activates NF-κB. Surprisingly, the mRNA levels of NF-κB p65 and p50 remain unchanged despite FOSL1 overexpression (Fig. 5F), indicating that mechanistic regulation at the transcriptional level was not involved. Given the observed increase in phosphorylated NF-κB (S536) levels while total NF-κB remained unchanged (Fig. 5G), we proceed to explore the potential involvement of post-translational protein regulation. To this end, after overexpressing FOSL1 in glioma cell lines A172, U87MG, and PDX-L14, we conducted Western blot analysis to assess upstream regulators of NF-κB, namely IKKβ and IκBα. The results revealed a significant increase in phosphorylated IKKβ (Ser176/Ser180) and IκBα (Ser32/Ser36) upon FOSL1 overexpression compared to the control in each cell line, while total IKKβ and IκBα remained unchanged (Fig. 5G). These results supported the hypothesis that FOSL1 activates NF-κB via the activated IKKβ kinase, which in turn triggers the activation of IκBα and subsequently initiates NF-κB signaling.

**Fig. 6:**
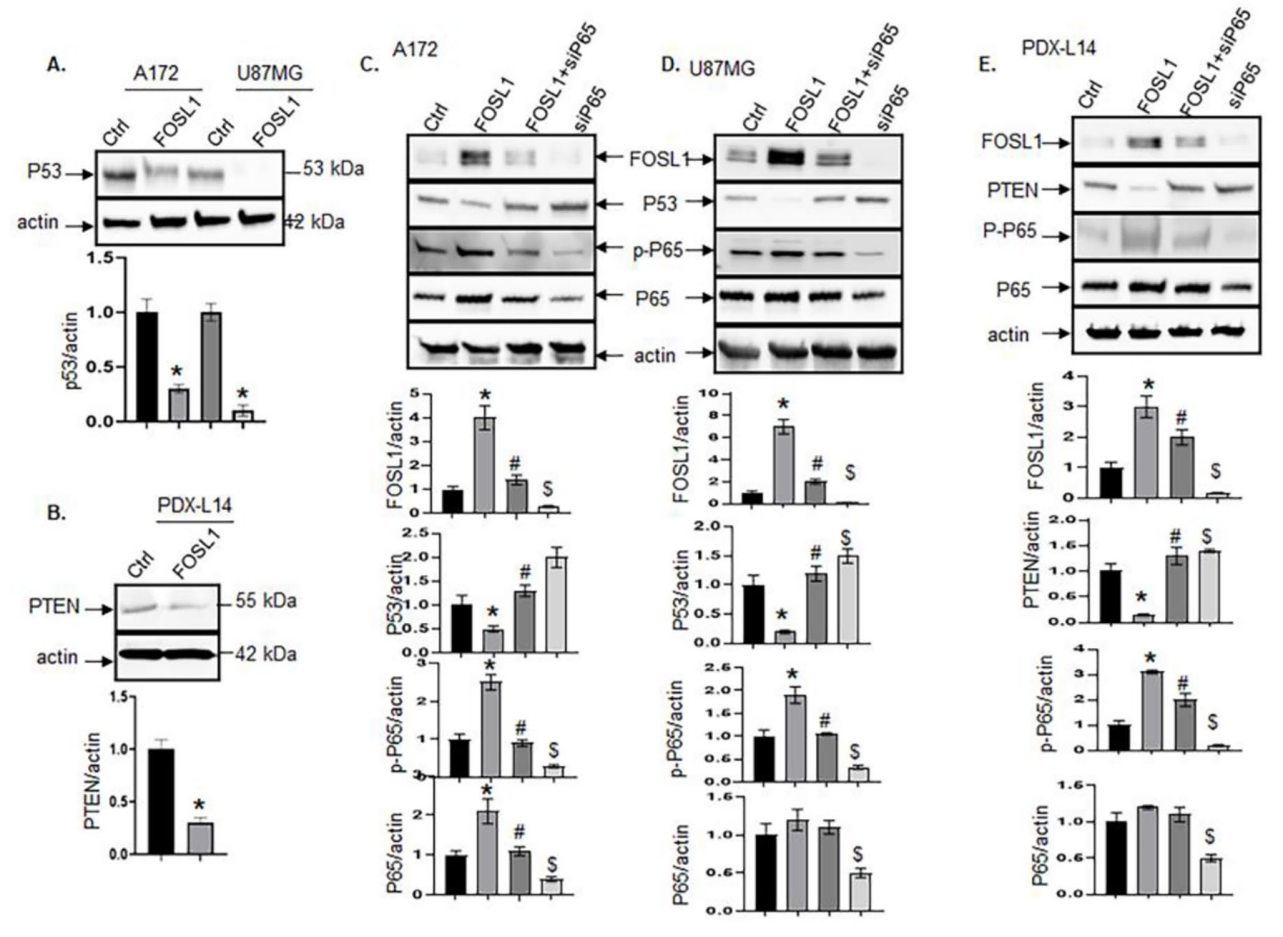
Overexpression of FOSL1 in GBM negatively correlates with tumor suppressors p53 and PTEN through directly activating NF-κB. (A, B) The tumor suppressors p53 (**A**) and PTEN (**B**) were examined when FOSL1 was overexpressed. (**C-D**) Upon reduced activation of P65 by siP65, inhibition of p53 by FOSL1 could be partially rescued in A172 (**C**) and U87MG (**D**), the inhibition of PTEN by FOSL1 could be partially rescued in PDX-L14 cells (**E**)

The Western blot results demonstrated that the overexpression of FOSL1 led to a reduction in p53 expression levels in A172 and U87MG cells (Fig. 6A), and a decrease in PTEN expression levels in PDX-L14 cells (Fig. 6B). To discern whether the effects induced by FOSL1 are direct or indirect effects, we transfected glioma cells with FOSL1 along with siRNA targeting NF-κB p65 (siNF-κB p65 or siP65) and corresponding controls. The outcomes revealed that upon reduced activation of p65 by siP65, inhibition of p53 by FOSL1 could be partially rescued in A172 (Fig. 6C) and U87MG (Fig. 6D) cells (as observed by comparing the 2nd and 3rd lanes on the blot and the densitometric measurements in the bar graph below the Western blot). Similarly, the inhibition of PTEN by FOSL1 could be partially rescued in PDX-L14 (Fig. 6E) cells (as evident from the comparison of the 2nd and 3rd lanes on the Western blot and the densitometric measurements in the bar graph below the blot). Our data indicate that in glioma cell lines expressing wild type p53 (wtp53, such as A172 and U87MG cells) and/or PTEN (wtPTEN, such as PDX-L14), FOSL1 positively influenced NF-κB while diminishing the protein expressions of p53 and PTEN, thereby directly activating of NF-κB. These findings also imply a bidirectional regulatory relationship between NF-κB and FOSL1 in gliomagenesis.

### GSC markers, CD133 and ALDH1, are positively associated with FOSL1 in GBM

**Fig. 7:**
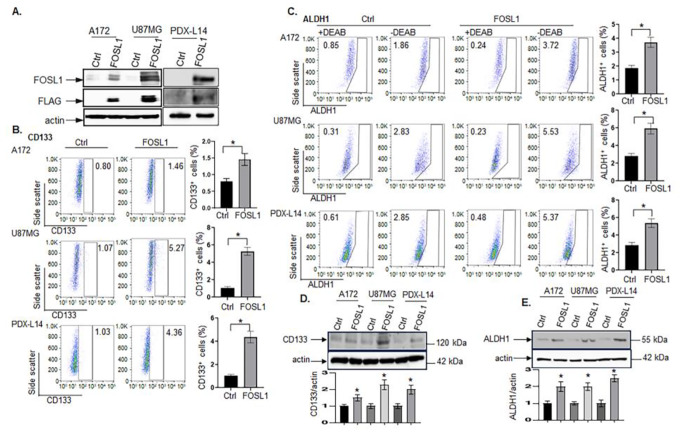
GSC markers, CD133 and ALDH1, are positively associated with FOSL1 in GBM. (**A**) A172, U87MG, and PDX-L14 cells were transfected with Flag-tagged human FOSL1 and controls followed by assaying protein expression of FOSL1 and Flag by Western blot. (**B**) Flow cytometric analysis to measure CD 133 expression was performed in A172, U87MG, and PDX-L14 glioma cells. (**C**) ALDH1 enzymatic activities determined by the Aldefluor assay was performed in A172, U87MG, and PDX-L14 glioma cells. (**D-E**) The identical cells treated as described in (**A**) were underwent analysis for CD133 (**D**) and ALDH1 (**E**) via Western blotting. The lower panel of panel D and E are the densitometry analyses of the bands performed using the Image Quant program (*p < 0.05, Student *t*-test)

Our previous publication shows that FOSL1 knockdown reduces the expression of GSC markers CD133 and ALDH1 [3], which indicates that the downregulation of FOSL1 resulted in a reduced GSC population. Next, FOSL1 expression levels were elevated by introducing FOSL1 tagged with Myc-DDK/FLAG (FOSL1-FLAG) into glioma cells, we confirmed high transfection efficiency by observing the robust expression of FOSL1 and FLAG protein in A172, U87MG, and PDX-L14 cells (Fig. 7A). The number of CD133^+^ cells also increased in A172 (Fig. 7B top), U87MG (Fig. 7B middle), and PDX-L14 cells (Fig. 7B bottom), respectively. The elevation of CD133 protein levels resulting from overexpressing of FOSL1 protein in these cell lines was subsequently validated through Western blot analysis (Fig. 7D). To address the question of whether FOSL1 would affect ALDH1, another GSC marker, the ALDEFLUOR assay was performed on an identical model of glioma cells as we described before [18], in which FOSL1 was overexpressed by FOSL1-FLAG. Here, our results produced further evidence that the overexpression of FOSL1 increases ALDH1^+^ cells in all three of the glioma cell lines examined as shown in Fig. 7C top (A172), Fig. 7C middle (U87MG), and Fig. 7C bottom (PDX-L14), compared with corresponding controls. The upregulation of ALDH1 protein levels due to the overexpression of FOSL1 protein in these cell lines was additionally affirmed through Western blot analysis (Fig. 7E). These combined findings suggest a positive correlation between the GSC markers CD133 and ALDH1 with FOSL1 in GBM, indicating that FOSL1 causatively induced the expression of GSC markers, CD133 and ALDH1, in GBM.

### FOSL1 stimulates the growth of glioma cells by preventing apoptosis and promoting the transition of cells from the S phase to the G2/M phase

**Fig. 8:**
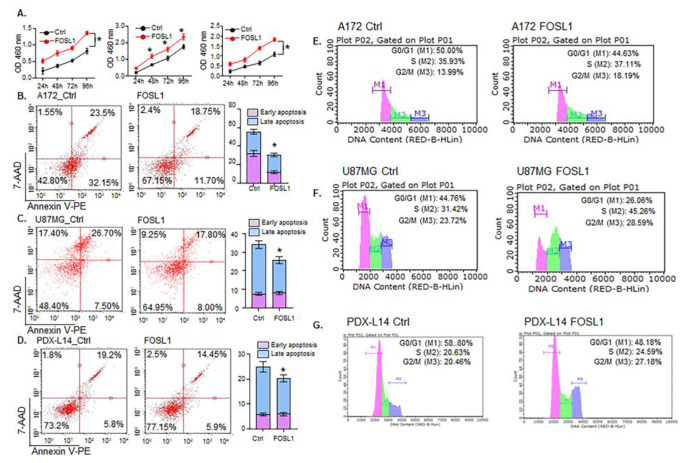
FOSL1 drives glioma cell growth and proliferation of glioma cells by inhibiting apoptosis and promoting the transition from the S to G2/M phase. (**A**) A172, U87MG, and PDX-L14 cells were transfected with Flag-tagged human FOSL1 along with controls for 24 to 96 h, followed by a CCK-8 assay to measure cell viability. (**B-D**) Apoptosis levels were assessed using flow cytometry with Annexin V-PE staining upon FOSL1 overexpression in A172 (**B**), U87MG (**C**), and PDX-L14 (**D**) cells. (**E-G**) cell cycle progression was examined by flow cytometry, generating representative histograms illustrating the mean percentage of cells in G0/G1, S, and G2/M phases for A172 (**E**), U87MG (**F**), and PDX-L14 (**G**) cells

Our previous findings demonstrated that reducing FOSL1 expression through siFOSL1 effectively decreased glioma cell proliferation by 30.6 to 50.2% within 24 to 96 h post-transfection in A172, U87MG cells, and PDX-L14 cells [3]. In parallel, in this study, upon transient transfection of FOSL1 into these glioma cell lines, we observed an increase in cell proliferation, as measured by CCK-8, ranging from 25.0 to 60.5% across the same three glioma cell lines mentioned above (Fig. 8A). Tumor progression commonly involves apoptosis inhibition and to investigate the role of the apoptotic process in GBM cells overexpressing FOSL1, glioma cells were transfected with FOSL1 for 72 h, followed by staining with annexin V and 7-AAD. Annexin V-FACS analysis was then performed to identify apoptotic cell death. The levels of early and late apoptosis were quantified as the percentage of cells positive for annexin V-PE without or with 7-AAD staining, respectively. The data revealed that heightened FOSL1 reduced the early apoptosis from 32.15 to 11.70% and late apoptosis from 23.5 to 18.75% in A172 cells (Fig. 8B). Similarly, increased FOSL1 led to a decrease in late apoptosis from 26.7 to 17.8% (Fig. 8C) in U87MG and 19.2 to 14.45% in PDX-14 cells (Fig. 8D). To delve deeper into the role of FOSL1 in driving proliferation, we investigated its potential to disrupt cell cycle distribution. We assessed the proportion of cells in G0/G1, S, and G2/M phases based on DNA content. The representative histograms in Fig. 8E revealed that heightened FOSL1 expression significantly altered the cell cycle distribution. This was evident in the increased percentage of cells in S phase (35.93 to 37.11%) and G2/M phase (13.99 to 18.19%), accompanied by a concurrent decrease in G0/G1 phase cells (50.00 to 44.63%) compared to the control group in A172 cells. Similarly, in U87MG cells, FOSL1 treatment led to a significant increase in the percentage of cells in the S phase from 31.42 to 45.26% and in the G2/M phase from 23.72 to 28.59%, while reducing G0/G1 phase cells from 44.76 to 26.06% (Fig. 8F). In PDX-14 cells, heightened FOSL1 expression significantly enhanced the percentage of cells in S phase from 20.63 to 24.59% and G2/M phase from 20.46 to 27.18%, while decreasing G0/G1 phase cells from 58.8 to 48.18% (Fig. 8G). These findings indicate that FOSL1 overexpression specifically disrupts the accumulation of cells in the G0/G1 cell cycle phase. Therefore, the functional analyses employing flow cytometry suggest that FOSL1 serves as an oncotarget in GBM, stimulating the growth and proliferation of glioma cells possibly by inhibiting cell apoptosis and promoting their cell entry into the S and G2/M phases of the cell cycle.

## Discussion

In this study, we delved into the interaction between NF-κB and FOSL1, revealing their role in driving and propelling glioma stemness and gliomagenesis. Our transcriptome and in vitro data revealed significant insights into FOSL1’s orchestration of the NF-κB signaling pathway, enhancing NF-κB’s activity while reducing the protein levels of p53 and PTEN protein in glioma cell lines featuring wild type p53 (wtp53) and/or PTEN (wtPTEN). Simultaneously, NF-κB triggers FOSL1 transcriptional activation in gliomagenesis. Both our in vitro experiments and clinical observations substantiate a direct correlation between NF-κB protein expression and FOSL1 levels in gliomas. Moreover, FOSL1 exhibits a positive correlation with GSC markers CD133 and ALDH1 in GBM. Functionally, FOSL1 promotes glioma cell proliferation by impeding apoptosis and facilitating cell transition from the S phase to the G2/M phase.

It has long been established that NF-κB and STAT3 signaling are closely intertwined and that they collaborate in a variety of pathological processes, with cancer being a notable example. Determining the precise interactions between these two precancerous pathways in GBM has, however, advanced significantly in recent years [35]. The most common mechanisms by which STAT3 and NF-κB transcriptional activities are induced and functionally interact with each other are (1) the members of NF-κB, such as RelA/p65, can physiologically interact with STAT3, and their association can modify their transcriptional activity [16, 36]; (2) as two important transcriptional factors, STAT3 and NF-κB cooperatively bind at a subset of gene promoters to collaboratively induce their target genes’ expression [15]; (3) many cytokines induced by STAT3 and NF-κB can feedback to induce NF-κB and STAT3 activation [37, 38]. Previously, we observed that STAT3 activates the FOSL1 gene, amplifying GBM stemness [3]. This prompted us to delve deeper into NF-κB’s role in transactivating FOSL1 during gliomagenesis in this study.

GBMs resistance to treatment and its challenging prognosis is mostly owing to the high fraction of GSC and the resulting intra-tumor heterogeneity from genetic, epigenetic, developmental, and microenvironmental factors [39]. Research shows that GSCs are essential for tumor initiation, invasion, angiogenesis, immune suppression, microenvironment maintenance, and treatment resistance. Therefore, exploring the molecular pathways that trigger GSC proliferation and tumor growth has the potential to offer new insights into the aggressive nature of GBM and uncover novel therapeutic targets [39].

A recent comprehensive approach utilizing single-cell transcriptome sequencing (scRNA-seq) has unveiled distinct categorizations with GBM cells, encompassing both adult and pediatric GBM. These cells delineate into four distinctive groups based on their gene expression profiles: neural-progenitor-like (NPC-like), oligodendrocyte-progenitor-like (OPC-like), astrocyte-like (AC-like), and mesenchymal-like (MES-like) states [40, 41]. Specifically, within the TCGA classification, the TCGA-PN subtype manifests as a blend of two distinct cellular states-OpC-like and NPC-like, while TCGA-CL aligns with AC-like characteristics. TCGA-MES, on the other hand, is characterized by a fusion of MES-like traits along with a dominance of microglia and macrophages. Moreover, TCGA tumors exhibiting heightened genetic amplifications of *EGFR* show a notable association with elevated AC-like bulk scores. Similarly, amplifications of *PDGFRA* and *CDK4* are linked respectively to OPC-like and NPC-like scores, consistent with their established roles as regulators of OPC and NPC in normal developmental processes. Additionally, alterations in Chr5q and *NF1* are correlated with MES-like cells. It is crucial to note that each tumor comprises of cells in multiple cellular states, capable of proliferation or transitioning to alternative states within this spectrum [41]. In our current study, the IDH1 wild type A172 GBM line (expressing TP53 and EGFR and bearing mutations in *PTEN* and *CDKN2A*/*p16INK4a*) [42, 43] and U87MG (expressing TP53 with *PTEN* mutations, *CDKN2C*/*p18INK4C* mutations, and O6-methylguanine-DNA methyltransferase (*MGMT*) promoter methylation) [42, 44] are employed as models akin to the AC-like and MES-like subtypes, respectively. Additionally, PDX-L14 cells representing the PN-like subtype showcase wild-type genes such as *PTEN*, *EGFR*, *CDKN2A*, *NF-κB*, *CDK4/MDM2*, an amplified gene *CSNK2A* gene, *TP53* deletion, and expression of CD133^+ 3,4^. When all three lines were subjected to FOSL1 overexpression, there was a significant increase observed in GSC markers CD133 and ALDH1 (Fig. 7). This observation strongly suggests that FOSL1 plays a role in inducing GSC stemness [3]. This finding aligns with previous studies, both from our research and other search groups, indicating FOSL1’s role in promoting the transition to the MES subtype [3, 23, 45].

Regarding NF-κB signaling, the most abundant form, the p65/p50 heterodimer, is called the canonical pathway [46, 47], where IκB is phosphorylated and degraded, leading to NF-κB release and translocation into the nucleus [48]. In the non-canonical pathway, p52/RelB heterodimer is induced and translocated into the nucleus. NF-κB regulates multiple key genes for cell proliferation and survival as well as the innate and adaptive immunities [48]. NF-*κ*B is activated by a number of factors, such as pro-inflammatory cytokines, interleukin-1*β*, bacteria, lipopolysaccharides (LPS), epidermal growth factor, T- and B-cell mitogens, viral proteins, physical and chemical stressors, and double-stranded RNA [49, 50]. Cellular stressors, such as being targeted by chemotherapeutic agents and ionizing radiation, also activate NF-*κ*B [51]. NF-*κ*B contributes to pro-tumor inflammation [52] and tumor angiogenesis [53] and is activated in a variety of tumors [47, 49, 52]. Chen et al. discovered that FOSL1 stimulates CYLD SUMOylation, relying on UBC9. Subsequently, this process leads to K63-linked polyubiquitination of major NF-κB intermediates, thereby initiating NF-κB activation. Consequently, this activation prompts PMT induction in GSCs [45]. The aberration of the NF-κB pathway is a characteristic that defines MES GBM. In addition, the transition to MES GBM as a response to therapy is also marked by increased activation of NF-κB signaling [35]. Our discoveries highlighted the pivotal role of NF-κB in triggering the transcriptional activation of FOSL1 in GBM stemness (Figs. 3 and 4).

FOSL1 functions as an AP-1 transcription factor involved in glioma pathogenesis. The AP-1 complex results from dimerization between members of JUN (c-Jun, JunB, and JunD) and FOS (c-Fos, FosB, Fra-1, and Fra-2) families [54]. FOSL1, encoding FRA-1, is overexpressed in most solid tumors including lung cancer [55], breast cancer [56], ovarian cancer [57], prostate cancer [58], gastric cancer [59], colorectal cancer [60, 61], head and neck squamous cell carcinomas [62], and GBM [45, 63]. The oncoprotein FOSL1’s overexpression is correlated with tumor progression and worsened patient survival [64–67]. Therefore, FOSL1 has emerged as a prominent therapeutic target [68]. FOSL1 protein is localized in the nucleus and cytoplasm [69]. This protein plays an crucial role in various aspects of cancer biology, including cancer cell proliferation, invasion/metastasis [4], epithelial-to-mesenchymal transition (EMT, is often related with acquisition of stemness characteristics [60, 70, 71]), cancer stemness [72, 73] and chemo-resistance [72], and antitumor immunity [74]. Particularly, FOSL1 promotes PN-to-MES transition of GSC [23, 45]. Mechanistically, FOSL1 undergoes regulation at multiple levels, encompassing transcriptional control by STAT3 and post-translational modifications, including deacetylation and phosphorylation, as observed in our research and other research groups [3, 69, 73]. . Our current study builds upon our earlier findings, uncovering that FOSL1 is activated not only by STAT3 but also by NF-κB within the context of GBM (Figs. 3 and 4). Our data indicates that FOSL1 facilitates the proliferation of GBM cells by inhibiting apoptosis and boosting cell transition from the S phase to G2/M phases in the cell cycle (Fig. 8).

The scRNA-seq data from four different cancer cell lines (A549, DU145, MCF7, and OVCA420) revealed that signaling pathways known to drive EMT- like TGFβ1, EGF, and TNF- converge on NF-κB and FOSL1, before initiating the expression of typical EMT master regulators such as STAIL, TWIST, and ZEB [75]. Likewise, NF-κB and FOSL1 are both pivotal transcription factors responsible for driving the mesenchymal state of GSCs. A notable discovery in the current study is the identification of mutual regulation between NF-κB and FOSL1 within GBM. On one hand, NF-κB triggers the activation of FOSL1 (Figs. 3 and 4), and on the other hand, FOSL1 influences NF-κB regulation, as evidenced by both RNA-seq data (Fig. 1) and in vitro experiments (Figs. 5 and 6).

The diverse nature of GBM is the reason that treatments in which heavily focused on a single molecule or pathway are mostly ineffective. To address the various subtypes found in each patient, it is necessary to target multiple molecular targets simultaneously. Simultaneously targeting both NF-κB and FOSL1 could potentially enhance therapeutic efficacy. In the future, it is imperative to initiate clinical trials to assess combination therapies that target certain subtypes and determine the most effective treatment protocols for patients.

### Electronic supplementary material

Below is the link to the electronic supplementary material.

Supplementary Material 1

Supplementary Material 2

## Data Availability

Data generated during the study are subject to a data sharing mandate and available in a public repository that does not issue datasets with DOIs.
