# Supplementary material for: Interaction of NF-κB and FOSL1 drives glioma stemness
Source: Cell Mol Life Sci. 2024 Jun 10;81(1):255. doi: 10.1007/s00018-024-05293-1 (PMC11335291; doi:10.1007/s00018-024-05293-1)
Supplement: Supplementary file 1 — Supplementary Material 1 [file 18_2024_5293_MOESM1_ESM.docx]

Supplementary documents


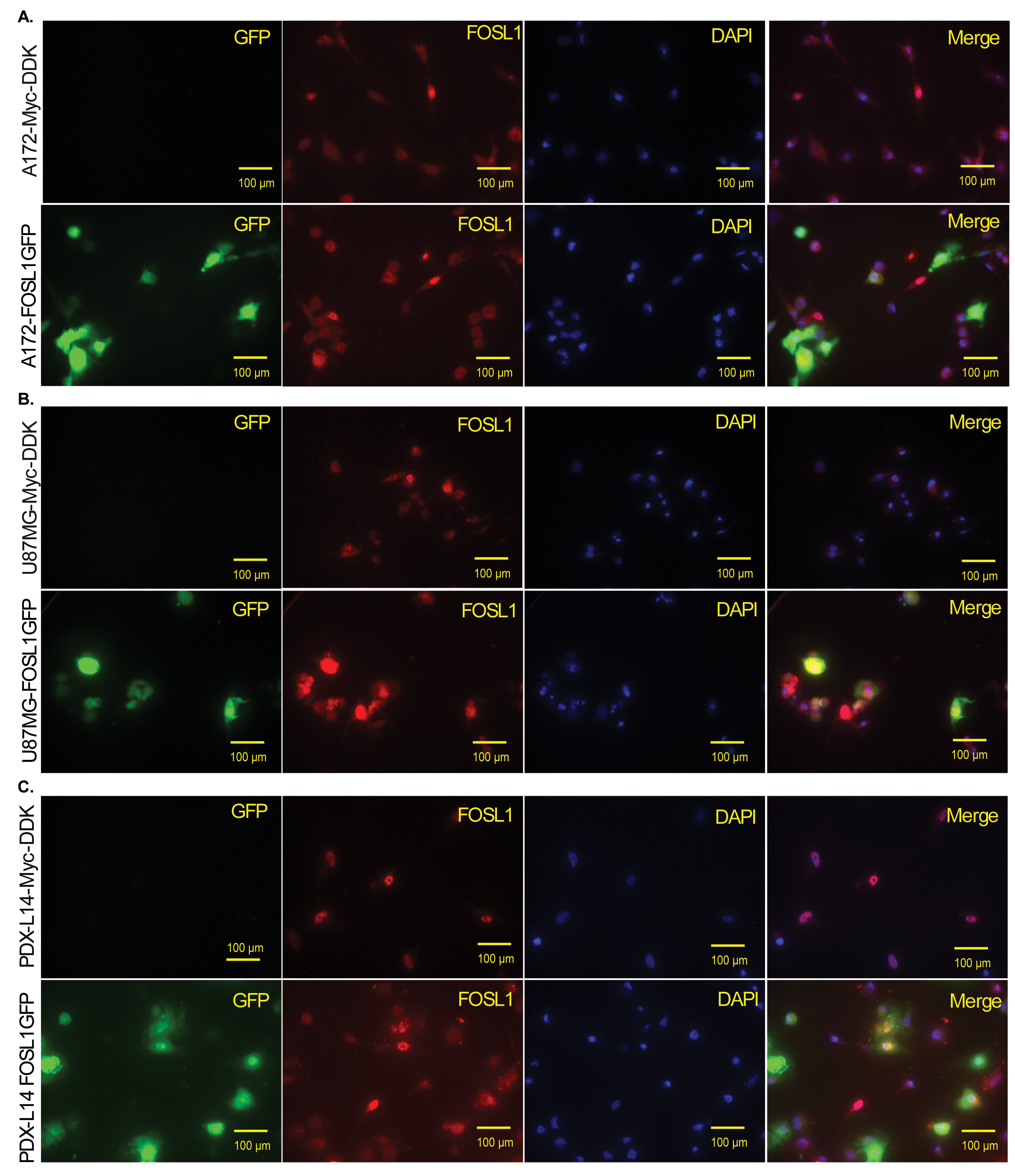
Supplementary Fig 1. GFP does not influence FOSL1 localization. (A). A172 cells transfected with a vector tagged with Myc-DDK and the identical vector with GFP-tagged FOSL1 for 72 h were stained for FOSL1; Magnification 40x. (B). U87 MG cells transfected with a vector tagged with Myc-DDK and the identical vector with GFP-tagged FOSL1 for 72 h were stained for FOSL1; Magnification 40x. (C). PDX-L14 cells transfected with a vector tagged with Myc-DDK and the identical vector with GFP-tagged FOSL1 for 72 h were stained for FOSL1; Magnification 40x.

Supplementary Fig 2. GFP does not influence FOSL1 function. Both GFP-tagged FOSL1 and Myc-DDK-tagged FOSL1, using the same backbone vector, were transfected into glioma cells A172, U87MG, and PDX-L14. Subsequently, qPCR analysis was performed to assess the mRNA expression of FOSL1 target genes and GSC markers CD133 (A) and ALDH1 (B).


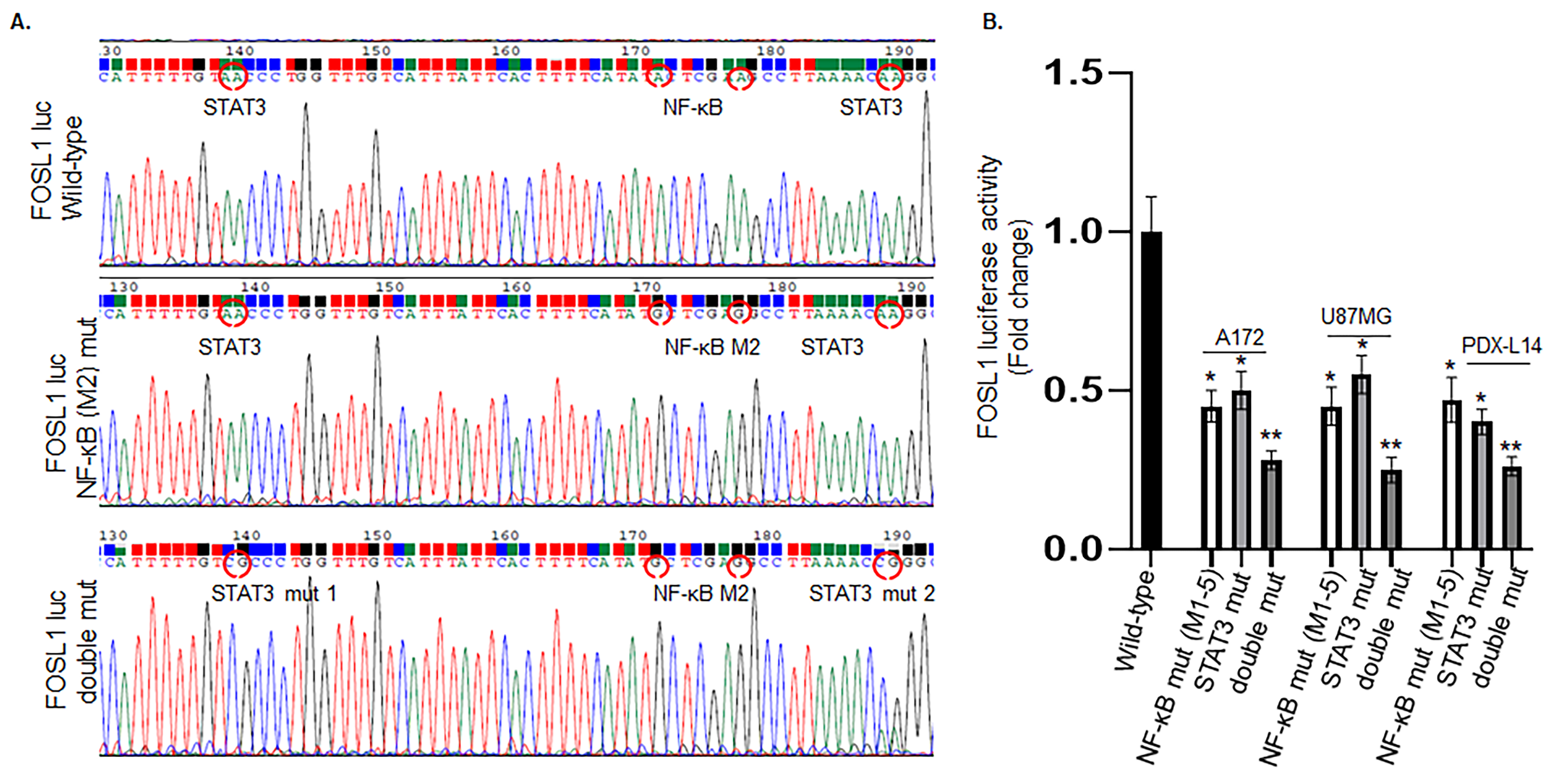


Supplementary Fig 3. Interplay and synergistic effects between STAT3 and NF-κB on FOSL1 promoter activity. (A) Partial sequences of the wild-type FOSL1 promoter (top), the NFκB binding site mutant M2 (middle), and the double mutant with mutations in both the STAT3 and NF-κB binding sites (bottom). For clarity and simplicity, only the sequence portion of the NF-κB binding site mutation (M2) and STAT3 binding site mutation were presented for the double mutant in Supplementary Fig 3A. (B) In A172, U87MG, and PDX-L14 cells, the double mutant exhibited a greater inhibition of FOSL1 transcriptional activity compared to individual mutations in STAT3 and NF-κB. *p<0.05, **p<0.01,
